# What Turns Task Crafters into High Performers? Affective Commitment and Strategic Alignment as Critical Levers

**DOI:** 10.3390/bs15050678

**Published:** 2025-05-15

**Authors:** Inyong Shin, Jong Gyu Park, Heesun Chae

**Affiliations:** 1 Division of Business Administration, Pukyong National University, Busan 48513, Republic of Korea; shiny@pknu.ac.kr; 2 College of Staten Island, City University of New York, New York, NY 10314, USA; jonggyu.park@csi.cuny.edu

**Keywords:** task crafting, affective commitment, strategic alignment, task performance

## Abstract

Considering that the impacts of task crafting on task performance are not uniform and may depend on goal congruence, this study attempted to explore the roles of affective commitment in individual–organizational goal congruence and strategic alignment in job–organizational goal congruence. Drawing on conservation of resources theory, we anticipated that affective commitment as a motivational resource and strategic alignment as an organizational resource would be critical levers for task crafting to affect task performance. Using a time-lagged design with two data-collection points, we conducted a multilevel analysis of data from 138 subordinates and 50 supervisors. As a result, we found that task crafting had the strongest positive effect on task performance when both affective commitment and strategic alignment were high. This study offers new insights regarding task crafting by identifying how employees can be effectively proactive. It also expands the theoretical application of conservation of resources theory by specifying how different types of resources interact to improve performance.

## 1. Introduction

In recent decades, job crafting, the proactive changes by which employees shape their jobs without requiring formal organizational approval, has emerged as an important concept in organizational behavior research (Holman et al., 2024; Wrzesniewski & Dutton, 2001). However, the notion that employees may actively reshape their own job roles is not new. Even proponents of the job characteristics model, which traditionally emphasizes top-down job design, have acknowledged that employees play a role in reshaping their tasks as part of the job redesign process (Kulik et al., 1987; Scharp et al., 2023). The growing ambiguity and volatility of today’s business environment has led organizations to increasingly rely on employee-driven job crafting, making this concept more relevant than ever (Boehnlein & Baum, 2022; Lee & Lee, 2018).

Among job crafting’s various forms, researchers have identified task crafting—where employees proactively modify the number, scope, or type of their tasks—as particularly influential (Holman et al., 2024; Leana et al., 2009; Lin et al., 2017; Wrzesniewski & Dutton, 2001). Employees engage in task crafting to better align their work with their personal strengths, preferences, and goals, often leading to increased skill utilization and reduced inefficiencies (Oldham & Hackman, 2010; Olafsen et al., 2025). As a self-initiated process, task crafting primarily involves employees themselves, allowing them to reshape their roles in ways that foster greater autonomy and job fit (Niessen et al., 2016). Consequently, task crafters tend to experience greater affective well-being and fulfillment of intrinsic needs (Slemp & Vella-Brodrick, 2013, 2014).

Now, researchers are interested in the organizational effectiveness of task crafting beyond its individual psychological benefits. Since task performance or in-role behavior represents the behavior of organizational members that directly supports organizational functions by fulfilling their core job responsibilities (Williams & Anderson, 1991), their attention is focused on how task crafting affects task performance. However, the evidence on how task crafting relates to task performance is far less uniform (Bizzi, 2017; Boehnlein & Baum, 2022). While some studies have found that task crafting enhances organizational effectiveness by fostering productivity (e.g., Geldenhuys et al., 2021; Weseler & Niessen, 2016; Yepes-Baldó et al., 2018), others have failed to establish a significant link between task crafting and task performance (e.g., Bizzi, 2017; Leana et al., 2009). There are even researchers who argue that task crafting may be detrimental to the organization (e.g., G.-N. Kim & Lee, 2016; Lee & Lee, 2018). Consequently, these inconsistent findings indicate that task crafting’s impacts on performance are not uniform and may depend on situational factors. Indeed, a meta-analytic study based on previous findings has revealed that the effect of task crafting on in-role performance varies across societal cultures (Boehnlein & Baum, 2022).

Scholars who first introduced the concept of task crafting argued that aligning individual work patterns with organizational objectives could be a net positive for the organization (Wrzesniewski & Dutton, 2001). In the same vein, researchers have suggested that task crafting would be beneficial to the organization when employees craft their tasks in ways that align with organizational goals (e.g., Grant & Parker, 2009; Lee & Lee, 2018). Surprisingly, however, no studies are known to have empirically investigated the role of goal congruence in examining the impact of task crafting on task performance. In this study, we attempt to address this research gap by exploring two factors that specify the congruence with organizational goals, from both personal and job perspectives. Specifically, we first focus on affective commitment, which reflects individual–organizational goal congruence (Meyer et al., 1993; Mowday et al., 1982). Next, we pay attention to strategic alignment, which captures task–organizational goal congruence (Biggs et al., 2014; Raper et al., 2020).

This study aims to provide a nuanced understanding of how task crafting relates to task performance by identifying the roles of affective commitment and strategic alignment in the relationship. Specifically, we sequentially examine the moderating effects of affective commitment and strategic alignment, with the expectation that how task crafting affects task performance depends on personal and job congruence with organizational goals. To accomplish this, we develop a theoretical model based on conservation of resources theory (Hobfoll, 1989; Hobfoll & Freedy, 1993), which explains how employees invest and leverage resources to optimize their work outcomes. In addition, we collect multi-source and time-separated data to reduce common method variance and increase the reliability of analytic results. Further, the model is validated using hierarchical linear modeling (HLM) to account for the nested nature of the data, based on the variable-centered approach focusing on the associations between variables (Howard & Hoffman, 2018). Finally, we discuss the theoretical contributions and practical implications of our findings.

This current research contributes to the task crafting literature by addressing inconsistencies about the effect of task crafting on task performance and uncovering how affective commitment and strategic alignment serve as important resources in determining the effectiveness of task crafting. It also expands the theoretical application of conservation of resources theory by specifying how different types of resources interact to improve performance.

## 2. Theoretical Framework and Hypothesis Development

### 2.1. Theoretical Framework

Recognizing that goal congruence is the key condition determining the relationship between task crafting and task performance, this study focuses on affective commitment in terms of individual–organizational goal congruence and strategic alignment in terms of task–organizational goal congruence. Clarifying the roles of affective commitment and strategic alignment requires a robust theoretical framework. In this study, we propose the theoretical framework based on conservation of resources theory (Hobfoll, 1989; Hobfoll & Freedy, 1993). At the core of the theory is the concept of resources, which are objects, conditions, characteristics, or energy that are considered valuable and a means of achieving centrally valued purposes (Hobfoll, 1988, 2002). In this framework, affective commitment functions as a motivational resource that energizes employees to engage in task crafting for the purpose of contributing to the organization, while strategic alignment serves as an organizational resource that provides the necessary structure and information to ensure that tasks align with broader organizational goals (Nguyen et al., 2016; Biggs et al., 2014).

Although scholars originally used conservation of resources theory to explain resource depletion, they have increasingly applied it to understand resource accumulation and gain (Halbesleben et al., 2014; Hobfoll et al., 2018). According to the theory, individuals should first invest resources in order to obtain new resources (Hobfoll & Freedy, 1993). This principle is suitable for discussing how affective commitment as a motivational resource interacts with task crafting to predict task performance. It also suggests that resource-rich individuals are better positioned to invest those resources in ways that maximize returns (Hobfoll, 2011). This principle is particularly relevant in understanding the role of strategic alignment as an organizational resource.

**Figure 1:**
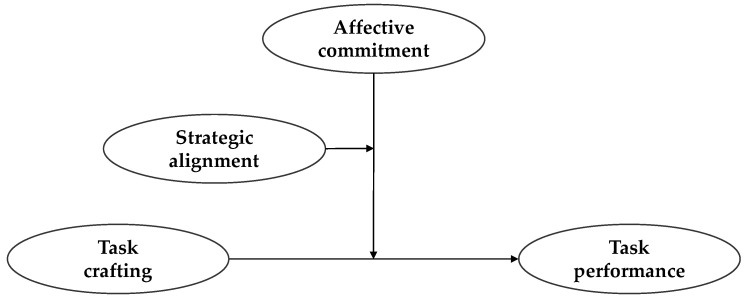
Conceptual model.

Building on these theoretical principles, we sequentially examine affective commitment and strategic alignment as the conditions under which task crafting enhances task performance. More specifically, we first investigate the joint effect of task crafting and affective commitment on task performance, and then the three-way interaction among task crafting, affective commitment, and strategic alignment. Figure 1 shows this study’s conceptual model and illustrates our hypotheses; below, we develop theoretical rationales for these hypotheses.

### 2.2. Affective Commitment as a First Lever

Researchers have identified sense of commitment as an important resource because it helps individuals achieve centrally valued ends (Hobfoll, 2002). In particular, affective commitment, which captures employees’ emotional involvement and identification with the organization (Meyer et al., 1993), functions as a motivational resource that enables employees to mobilize effort and energy to conduct their tasks (Mercurio, 2015; Nguyen et al., 2016). Employees with high affective commitment tend to have a strong emotional attachment to the organization, internalize the organization’s goals and values, and work to benefit the organization (Meyer et al., 1993, 2002; Mowday et al., 1982; K. Y. N. Ng, 2023). This suggests that affective commitment serves as both an attitudinal factor and a guiding force that influences how employees engage with their work, particularly in proactive job modifications like task crafting.

According to conservation of resources theory, individuals have to invest their resources to obtain additional resources (Hobfoll & Freedy, 1993). Applying this principle to affective commitment implies that employees with higher levels of affective commitment have already invested more resources in the organization (Halbesleben et al., 2008). In other words, affectively committed employees are those who have put their resources, such as attention, interest, and energy, into the organization. Further, the theory suggests that the more resources employees have invested, the more likely they are to attempt to increase the return on those resources (Halbesleben et al., 2008). These attempts by employees who are affectively committed to the organization are more likely to be expressed as actions that help them achieve the organization’s goals because achieving the organization’s goals returns new resources to them, such as performance-based pay, promotions, job security, and career growth. Indeed, research has shown that affectively committed employees strive to obtain work outcomes that they perceive as valuable to the organization (Mercurio, 2015; Meyer et al., 2002; K. Y. N. Ng, 2023).

Given that affectively committed employees tend to behave in the best interests of their organization (Luu, 2018), it seems that affective commitment encourages employees to shape the direction of their crafting efforts. Specifically, employees with high affective commitment are more likely to modify their tasks in ways that optimize resources to improve the quality and efficiency of their work and take on additional responsibilities and quantities to align with organizational priorities. These behaviors contribute directly to the organization’s strategic goals and increase the employees’ likelihood of being recognized as a high performer (Grant et al., 2009). In contrast, employees with low affective commitment likely engage in task crafting primarily for self-serving reasons, such as need fulfillment or workload reduction, rather than considering their contributions to the organization.

Taken together, these arguments suggest that task crafting alone does not necessarily lead to higher performance; rather, its effectiveness depends on affective commitment, which means whether employees’ motivational resources are invested in the organization and whether they are aligned with the organization’s goals. Affectively committed employees are more likely to leverage their task crafting behaviors to benefit their organizations because they align themselves with the organization’s goals. In this study, we expect employees with high affective commitment to engage in task crafting that enhances their work efficiency and aligns with organizational objectives, thereby strengthening their positions to gain additional resources. Accordingly, we hypothesize the following:

**H1.**  *Task crafting will have a stronger positive effect on task performance when affective commitment is high.*

### 2.3. Strategic Alignment as a Second Lever

Strategic alignment refers to the connection employees perceive between their tasks and the organization’s strategic priorities; therefore, it encompasses their recognition of organizational objectives, understanding of their importance, and awareness of how daily tasks contribute to achieving them (Biggs et al., 2014; Raper et al., 2020). Ensuring that employees comprehend not only the organization’s overarching goals but also their specific duties, performance evaluation criteria, and expected contributions, strategic alignment allows them to better integrate their efforts into the broader organizational framework (J. Kim et al., 2020). Employees who experience strong strategic alignment tend to perceive their tasks as meaningful, goal-directed, and integral to organizational success, while boosts their engagement and performance (Biggs et al., 2014; Boswell, 2006).

Strategic alignment functions as an organizational resource in that it increases employees’ attachment to the organization, raises their interest in the organization’s strategic success, and provides them with the knowledge and capabilities to devote their energy into their daily work (Biggs et al., 2014; Boswell, 2006). According to conservation of resources theory, individuals with more resources are more advantageously positioned to invest resources, especially where the potential return on investment is highest (Halbesleben et al., 2014; Hobfoll & Freedy, 1993). This principle leads to the expectation that in addition to motivational resources, the influx of organizational resources will be both a resource pool and a driving force to maximize the return on investment.

While affective commitment motivates employees to craft their tasks within the scope of organizational goals, strategic alignment provides them with the contextual understanding necessary to ensure that crafted tasks align with the organization’s objectives and strategic priorities. Even if motivated employees may change their tasks independently and voluntarily, an accurate and broad understanding of the organizational environment needs to be accompanied to secure the sustainability of the change (Dutton et al., 2001). The awareness of the alignment between the organization’s strategic priorities and employees’ tasks can be used as appropriate contextual information for their proactive task-changing behavior, which can help gauge and identify the outcome of such behavior. Furthermore, when the tasks they have changed are judged to be well integrated and functioning smoothly within the organization, evaluators are more likely to respond favorably to them (Grant et al., 2009).

In this study, we propose that strategic alignment does not play a sole moderating role in the relationship between task crafting and task performance, but rather it complements affective commitment to enhance task performance together. When affective commitment and strategic alignment work together, task crafting is more likely to result in performance-enhancing behaviors than self-serving modifications that may not align with broader organizational objectives. Employees who perceive a strong fit between their tasks and organizational goals are better positioned to leverage both their motivation and contextual knowledge, enabling them to maximize the useful resources they can gain from the organization. Without this alignment, employees may struggle to assess the consequences of their task crafting efforts, leading to misdirected or inefficient modifications.

Overall, these arguments suggest that task crafting, affective commitment, and strategic alignment interact to shape task performance. Specifically, the strongest positive effect of task crafting on performance presumably occurs when employees have high affective commitment and high strategic alignment: such employees are not only motivated but also equipped with the necessary contextual understanding to align their crafted tasks with organizational goals. Accordingly, we hypothesize the following:

**H2.**  *Task crafting will have the strongest positive effect on task performance when both affective commitment and strategic alignment are high.*

## 3. Methodology

### 3.1. Sample and Data Collection

The data for this study was gathered through a field survey conducted across multiple industries in South Korea, including the manufacturing, finance, information technology, and service sectors. This study focused on full-time employees working in these industries. Before administering the survey, participants were informed about the study’s purpose and assured that their responses would remain confidential. Following the recommendations of Podsakoff et al. (2003), we employed a time-lagged and multi-source design to mitigate common method variance and enhance the reliability of our findings. Specifically, the survey process involved both subordinates and their immediate supervisors. Initially, subordinates completed surveys assessing the independent variable and moderating variables, including task crafting, affective commitment, and strategic alignment. After a six-month interval, their immediate supervisors were asked to evaluate their subordinates’ task performance.

A total of 150 dyads were surveyed, of which 145 provided valid responses, yielding a response rate of 93.3%. After excluding cases with incomplete supervisor data, mismatched identifiers, or timing inconsistencies between the two survey waves, 138 dyads (138 subordinates and 50 supervisors) were retained for the final analysis. According to established academic guidelines (Maas & Hox, 2005), this sample size was deemed sufficient for statistical analysis as it exceeded the recommended range of 125 to 250 dyads based on the number of measurement items used in this study (25 items in total).

The demographic characteristics of the final sample of 138 respondents are as follows: 55 participants (39.9%) were female, with an average age of 39.83 years (SD = 10.2) and an average tenure of 5.95 years (SD = 6.0). In terms of education, 29.7% of the participants had completed only a high school diploma, 21.7% had finished junior college, 40.6% had a bachelor’s degree, and 7.9% had a doctoral degree. Participants were employed across various industries, including the manufacturing (*n* = 33, 23.9%, SD = 10.96), finance (*n* = 27, 19.6%, SD = 7.40), information technology (*n* = 45, 32.6%, SD = 10.81), and service sectors (*n* = 33, 23.9%, SD = 9.93).

The normality of the data was assessed by examining the skewness and kurtosis values of all study variables. All skewness values were within the range of −1 to +1, and all kurtosis values were below an absolute value of 2, indicating no substantial deviation from normality (West et al., 1995). These results meet the commonly accepted thresholds for univariate normality (Podsakoff et al., 2003). Additionally, variance inflation factor (VIF) values were calculated to assess the potential for multicollinearity among the predictor variables. All VIFs ranged from 0.06 to 3.07, which falls well below the commonly accepted threshold of 5.0 (O’Brien, 2007), indicating that multicollinearity was not a threat to the validity of the coefficient estimates obtained from the HLM analysis.

### 3.2. Measures

All measures were rated on a 5-point Likert scale, ranging from 1 (strongly disagree) to 5 (strongly agree). The items were originally developed in English and translated into Korean using the standard back-translation method (Brislin, 1980).

Task crafting: Task crafting was assessed using six items from Slemp and Vella-Brodrick’s (2013) study. Sample items include “I voluntarily modify work procedures that I think are unproductive” and “I change the scope or types of tasks on my own”. The scale showed high reliability, with a Cronbach’s alpha of 0.91.

Affective commitment: To measure affective commitment, we utilized five items from Meyer et al. (1993). Sample items include “I would be very happy to spend the rest of my career with this organization” and “This organization has a great deal of personal meaning for me”. The reliability of this scale was also high, with a Cronbach’s alpha of 0.87.

Strategic alignment: Strategic alignment was measured using four items from Biggs et al. (2014). Example items include “I have a clear understanding of the organization’s strategic priorities” and “I am aware of how my day-to-day work aligns with the organization’s strategic priorities”. This scale had a Cronbach’s alpha of 0.91.

Task performance: Task performance was evaluated by the immediate supervisor using six items from Williams and Anderson (1991). Example items include “This employee adequately completes assigned duties” and “This employee meets formal performance requirements of the job”. The Cronbach’s alpha for this measure was 0.92.

Control variables: To avoid potential confounding effects on task performance, we included several control variables commonly used in prior organizational behavior research (e.g., Kamdar & Van Dyne, 2007; Srivastava et al., 2006). Specifically, we controlled for five variables: age, gender, educational level, and organizational tenure. These variables were selected because they are known to influence both employee behavior and performance outcomes and may obscure the unique effects of the focal predictors if not properly accounted for (Becker, 2005).

Age and organizational tenure were measured in years. Prior studies have reported that they are associated with task performance. For instance, Miraglia et al. (2017) found that both age and tenure were negatively related to in-role performance (*r* = −0.29 and −0.32, respectively). These effects may reflect decreased adaptability or misalignment with evolving job demands among older or long-tenured employees (T. W. H. Ng & Feldman, 2010). Gender was coded as 0 for female and 1 for male. Meta-analytic results have revealed that females tend to demonstrate higher task performance than males (Mackey et al., 2019). Educational level was coded as 1 = high school, 2 = junior college, 3 = bachelor’s degree, 4 = master’s degree, and 5 = doctoral degree. Higher educational attainment has been linked to better performance in cognitively demanding jobs (T. W. H. Ng & Feldman, 2009). Controlling for these demographic variables helped to isolate the effects of focal predictors on performance, thereby enhancing the internal validity of our model (Carlson & Wu, 2012).

### 3.3. Analytical Apporach

For the statistical analysis, we utilized SPSS 27 and AMOS 27 to conduct basic statistical tests, including confirmatory factor analysis, reliability tests, and correlation analysis. While all variables were initially analyzed at the individual level, we employed HLM to account for the interdependence of employees evaluated by the same supervisor (Raudenbush & Bryk, 2002). Supervisors rated the task performance of approximately 2 to 6 direct reports, with an average of 2.8 employees per supervisor. Given the hierarchical structure of the data, where employees were nested under supervisors, the between-group variance for task performance was found to be 27.8% (*p* < 0.001). The intraclass correlation coefficient (ICC1) for task performance was 0.28, which exceeds the commonly accepted threshold of 0.10 (Bliese, 2000), indicating sufficient between-group variance to justify multilevel analysis. This necessitated the use of multilevel analysis techniques like HLM to appropriately model the nested data structure.

To enhance the interpretability of the results, all predictor variables were group-mean centered following the recommendations of Hofmann and Gavin (1998). To examine interaction effects, we applied visualization techniques based on the approach proposed by Aiken and West (1991). Specifically, we plotted graphs illustrating moderation effects by calculating regression slopes at different levels of the moderator (mean ± 1SD). Additionally, we conducted *t*-tests to assess the significance of simple slopes at different levels of the moderator, providing a clearer understanding of how the relationships between variables varied across different conditions. To further probe the interaction effect, we also conducted a Johnson–Neyman analysis to identify the specific range of the moderator (affective commitment) for which the effect of task crafting on performance becomes statistically significant.

## 4. Results

### 4.1. Reliability and Validity Testing

We assessed the reliability of each variable, with Cronbach’s alpha coefficients for all variables measuring 0.8 or higher, indicating a high level of internal consistency (Nunally & Bernstein, 1978). Based on this, we concluded that the measurement items used in this study demonstrate strong reliability. Following the reliability analysis, we conducted confirmatory factor analysis (CFA) to examine the construct validity of the key variables. Model fit was evaluated using standard fit indices, where a comparative fit index (CFI) and incremental fit index (IFI) greater than 0.90, along with a root mean square error of approximation (RMSEA) below 0.08, indicate an acceptable model fit (Kline, 2005). As shown in Table 1, the hypothesized four-factor model demonstrated an excellent overall model fit: *χ*^2^ (182) = 296.11, *p* < 0.001, CFI = 0.95, IFI = 0.95, and RMSEA = 0.07. Additionally, the four-factor model fit the data better than any of the alternative models tested, further supporting the construct validity of the measurement model. The factor loadings of all items exceeded the recommended threshold of 0.50 and were statistically significant. We also applied the Fornell–Larcker criterion, confirming that the square root of the AVE for each construct was greater than its correlations with other constructs. It was thus judged that the variables included in this study had acceptable levels of convergent and discriminant validity (Fornell & Larcker, 1981; O’Leary-Kelly & Vokurka, 1998). Based on these CFA results, we proceeded with hypothesis testing using the four key variables included in the study.

### 4.2. Descriptive Statistics and Correlations

Table 2 presents the means, standard deviations, and correlations among the focal variables. The correlation analysis revealed that the independent variable task crafting did not show a statistically significant relationship with task performance (*r* = 0.00, n.s.).

### 4.3. Hypothesis Testing

Hypothesis 1 proposed that task crafting and affective commitment interact to affect task performance. To test this hypothesis, an HLM analysis was employed. As shown in Model 1 of Table 3, after controlling for the demographic variables, task crafting did not have a statistically significant direct effect on task performance (*γ* = 0.06, n.s.). However, as indicated in Model 2 of Table 3, task crafting and affective commitment interacted to positively predict task performance (*γ* = 0.17, *p* < 0.05).

**Figure 2:**
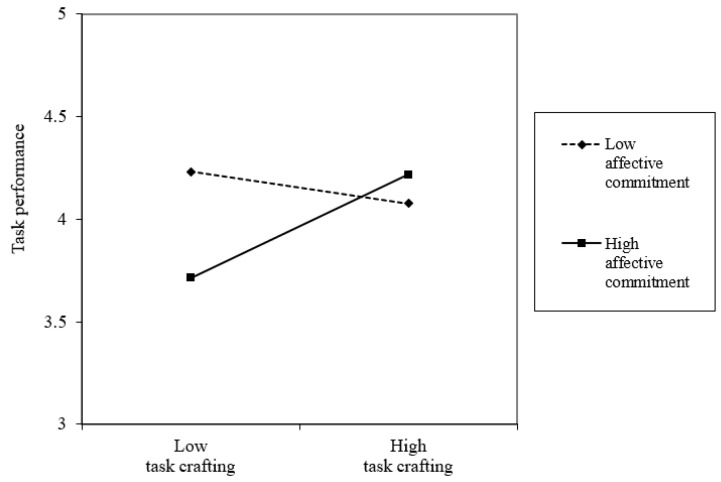
Two-way interaction of task crafting and affective commitment on task performance.

A simple slope analysis was conducted to further explore this interaction (Aiken & West, 1991). As shown in Figure 2, the positive relationship between task crafting and task performance was significant when affective commitment was high (*b* = 0.25, *p* < 0.05), but became insignificant when affective commitment was low (*b* = −0.08, n.s.).

**Figure 3:**
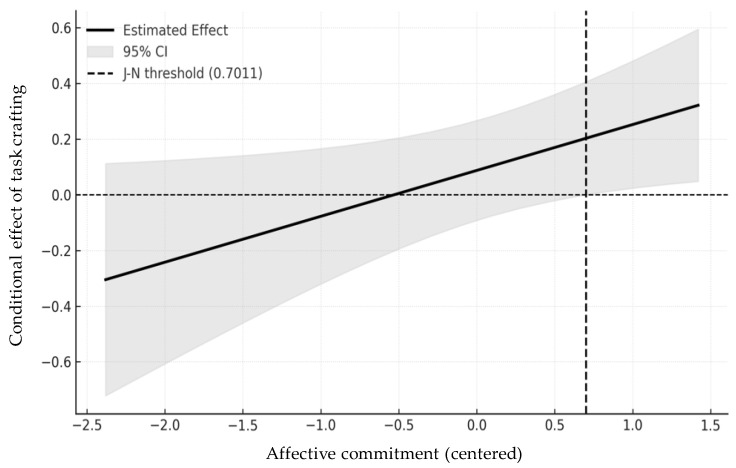
Johnson–Neyman plot.

In addition to the simple slopes analysis, the Johnson–Neyman technique was employed to more precisely identify the range of affective commitment values for which the effect of task crafting on performance is statistically significant. As depicted in Figure 3, the effect of task crafting becomes significant when affective commitment (centered) exceeds 0.70, which corresponds to approximately the top 16.7% of the sample. This result further confirms that the benefits of task crafting are contingent upon high levels of affective commitment. Therefore, Hypothesis 1, which expected that task crafting would have a stronger positive effect on task performance when affective commitment was high, was supported.

Hypothesis 2 proposed that task crafting, affective commitment, and strategic alignment interact to affect task performance. As shown in Model 5 of Table 3, the three-way interaction between task crafting, affective commitment, and strategic alignment significantly predicted task performance (*γ* = 0.19, *p* < 0.05). To provide a more rigorous and incremental test of our hypothesis, we additionally examined Model 3 of Table 3 to evaluate whether strategic alignment alone moderated the relationship between task crafting and performance. This analysis parallels Model 2 of Table 3, in which the moderating role of affective commitment was tested independently. The results indicated that the interaction between task crafting and strategic alignment was not statistically significant (*γ* = 0.15, n.s.), suggesting that strategic alignment by itself does not alter the effect of task crafting. However, the significant three-way interaction found in Model 5 of Table 3 implies that strategic alignment enhances the positive effect of task crafting only when accompanied by high affective commitment, reinforcing the importance of their combined influence.

**Figure 4:**
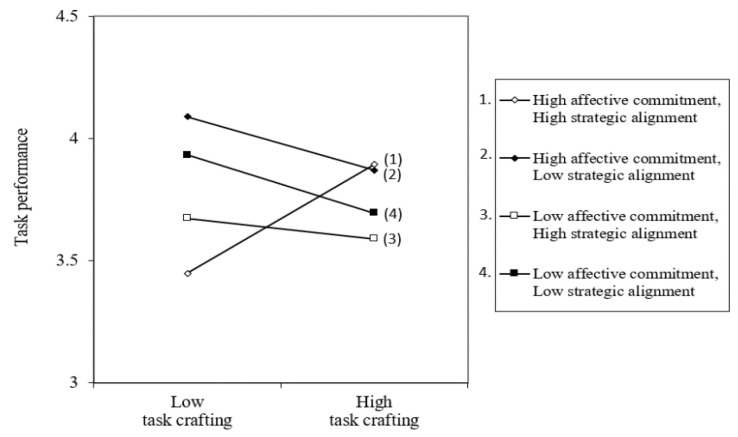
Three-way interaction of task crafting, affective commitment, and strategic alignment on task performance.

Figure 4 presents the results of a simple slope analysis to further examine this significant three-way interaction. When strategic alignment was high, affective commitment had a positive moderating effect on the relationship between task crafting and task performance, such that the relationship was significant when affective commitment was high (*b* = 0.55, *p* < 0.01) but not significant when affective commitment was low (*b* = −0.28, n.s.), as shown by Slopes 1 and 3 in Figure 4. Conversely, when strategic alignment was low, the relationship between task crafting and task performance did not vary significantly regardless of the level of affective commitment (*b* = −0.15, *b* = 0.19, both n.s.), as illustrated by Slopes 2 and 4 in Figure 4. These findings support Hypothesis 2, indicating that task crafting has the strongest positive effect on task performance when both affective commitment and strategic alignment are high.

## 5. Discussions

Previous findings regarding the relationship between task crafting and task performance have been inconclusive and a lack of understanding of whether task crafting benefits organizations has hindered both theoretical advancement and practical applications in this domain. To address this gap, this study examined the role of goal congruence in shaping the effectiveness of task crafting, focusing on affective commitment (individual–organizational goal congruence) and strategic alignment (task–organizational goal congruence) as key contextual factors. The analysis showed that task crafting alone does not directly predict task performance (*γ* = 0.06, n.s.), which is consistent with the findings from previous studies (e.g., Bizzi, 2017; Leana et al., 2009). Instead, its impact depends on employees’ affective commitment to the organization and strategic alignment with organizational goals. Specifically, the results revealed a joint effect of task crafting and affective commitment, as well as a three-way interaction involving task crafting, affective commitment, and strategic alignment. These findings reinforce the argument that task crafting is most beneficial when employees are affectively committed to their organization and when their tasks are aligned with strategic priorities. By highlighting the conditions under which task crafting enhances performance, this study provides valuable insights into how organizations can encourage proactive job redesigns that align with broader organizational objectives.

Meanwhile, in our study, gender was significantly and negatively correlated with task performance (*r* = −0.22, *p* < 0.01) and also emerged as a significant predictor in the HLM analysis (*γ* = −0.30, *p* < 0.01). A one-way ANOVA confirmed that female employees received higher performance ratings than male employees (*F*(1, 136) = 7.00, *p* = 0.01). This result aligns with prior findings suggesting that women may experience greater pressure to demonstrate competence in male-dominated environments or benefit from fairness-aware evaluation practices (Bowles, 2012; Joshi et al., 2015).

### 5.1. Theoretical Contributions

This present study makes several significant theoretical contributions. It advances the literature on task crafting by addressing the ongoing debate regarding its impact on task performance. While prior studies have often assumed that task crafting leads to positive outcomes, empirical findings have been inconsistent (e.g., Bizzi, 2017; Leana et al., 2009). Our study challenges the assumption that task crafting is universally beneficial, demonstrating that it enhances performance only under specific conditions—namely, affective commitment and strategic alignment. Notably, task crafting did not have a statistically significant direct effect on task performance, underscoring that task crafting alone does not necessarily lead to improved performance. Instead, its impact depends on whether employees are aligned with organizational goals in terms of personal and job aspects. By identifying these boundary conditions, our study offers a more nuanced understanding of how task crafting translates into organizational effectiveness. As such, it provides a context-sensitive framework for clarifying when and how task crafting contributes to organizational success. Furthermore, task crafting has been widely recognized as a proactive behavior that enables employees to take initiative in shaping their work environments (Grant & Parker, 2009). Considering that research on how to elicit wise proactivity has been requested (Parker et al., 2019), this study is significant in that it has discovered ways for employees to be proactive in an effective way.

Furthermore, this study elucidates how affective commitment and strategic alignment serve as significant resources in determining task crafting effectiveness. Affective commitment provides the motivation employees need to invest effort in crafting their task to align with organizational objectives (Meyer et al., 1993). Employees with high affective commitment are more likely to craft their tasks in ways that enhance work efficiency and contribute positively to the organization because they are emotionally invested in the organization’s success. Meanwhile, strategic alignment functions as an organizational resource that provides employees with the necessary contextual understanding of how their crafted tasks fit within the organization’s broader strategic priorities (Biggs et al., 2014). Interestingly, however, it has been found that strategic alignment alone does not contribute to increasing the effectiveness of task crafting, strengthening the moderating effect of affective commitment. This implies that while affective commitment serves as a motivator for employees to initiate task crafting behavior in a direction that benefits the organization, strategic alignment functions as a reinforcement to maintain such behavior. In other words, this study has demonstrated that both the necessary condition of effective commitment and the sufficient condition of strategic alignment should be met in order for task crafters to become high performers. It thus provides a more balanced and context-dependent framework for understanding task crafting’s impact on performance.

Next, this study expands the theoretical application of conservation of resources theory by specifying how different types of resources interact to shape task performance. Conservation of resources theory posits that individuals with greater access to valuable resources are more likely to invest resources in behaviors that yield further benefits (Hobfoll, 2002). This study refines this perspective by demonstrating that the effectiveness of task crafting depends on whether employees possess both motivational (affective commitment) and organizational (strategic alignment) resources. By identifying how these two distinct resource types interact to shape the impact of task crafting on performance, this study refines conservation of resources theory’s resource-based perspective on proactive work behaviors. These findings highlight the roles of relevant resource pools which have been relatively underexplored in conservation of resources theory-based research.

### 5.2. Practical Implications

This study provides important insights for organizations seeking to leverage task crafting as a strategic tool while ensuring its alignment with broader organizational goals. The finding that task crafting does not inherently improve performance but is contingent on affective commitment and strategic alignment indicates that organizations should actively create conditions that support these factors. Strengthening affective commitment ensures that employees are emotionally invested in their organization and motivated to contribute to organizational success; meanwhile, fostering strategic alignment helps employees understand how their tasks connect to their organization’s long-term objectives. By reinforcing these elements, organizations can maximize the positive effects of task crafting and prevent misalignment that can hinder overall effectiveness.

From a human resource management perspective, the findings of this study indicate that organizations should implement structured initiatives to support productive task crafting. Providing training and development programs that equip employees with the skills to modify their tasks in ways that align with organizational priorities can help them make informed decisions. Mentoring programs can be introduced to ensure that employees, particularly new hires or those in transitioning roles, receive guidance on how to engage in task crafting efforts that align with strategic goals. In addition, organizations should design performance management and reward systems to recognize not only the completion of assigned tasks but also proactive efforts to enhance work processes in ways that contribute to strategic objectives. Establishing cultures in which task crafting is acknowledged and rewarded can encourage employees to engage in meaningful job modifications.

By providing direction and ensuring that employees’ efforts align with organizational goals, management plays a pivotal role in facilitating effective task crafting. Managers should actively communicate their organization’s strategic priorities and provide employees with the contextual understanding needed to align their task modifications with these objectives. When leaders offer clear guidance and maintain open communication, employees are more likely to engage in task crafting that contributes to organizational effectiveness rather than focusing solely on personal interests. Additionally, leaders should engage in ongoing coaching, providing real-time feedback and individualized guidance to help employees refine their task crafting strategies and ensure that their efforts align with the evolving needs of their organization. High-quality leader–member relationships can foster trust and encourage employees to discuss potential task modifications with their supervisors, creating an open dialog for aligning personal and organizational goals.

To successfully embed task crafting within organizational frameworks, organizations should integrate affective commitment and strategic alignment into their policies and practices. To help ensure that employees understand how their contributions fit into the broader vision, organizations should clearly articulate their missions and values. They should also structure training and performance evaluation systems to reinforce these principles; this will help employees recognize the importance of aligning their work with strategic goals. Finally, leaders should act as facilitators who provide employees with both the autonomy to engage in task crafting and the necessary guidance to ensure that their proactive behaviors support organizational success.

By fostering work environments where organizational policies, human resource management practices, and leadership strategies actively support affective commitment and strategic alignment, organizations can maximize the benefits of task crafting. In short, organizations that ensure employees have both the motivation and contextual understanding to engage in task crafting effectively will be able to leverage proactive work behaviors. Nonetheless, these suggestions need to be flexibly applied according to the realistic circumstances of organizations. Depending on whether the strategic orientation of an organization is to improve the efficiency of existing operations or to pursue organizational changes based on individual innovative behavior, the organizational goals and individual performance standards may differ. In addition, the context of each organization, such as cultural resistance, hierarchical constraints, and unequal resources within the organization, may also be considered. Therefore, it is recommended that the organization applies our suggestions selectively or in stages rather than applying them all at once to suit the organizational situation.

### 5.3. Limitations and Future Research Directions

Despite the valuable insights this study provides, it has several noteworthy limitations that highlight avenues for future research. Firstly, although it employed a time-lagged research design with a six-month interval to reduce common method bias, collecting data from both employees and their direct supervisor, future studies could establish a more rigorous research framework that further minimized potential biases by using multiple sources (e.g., evaluations from both supervisors and colleagues) to construct the dependent variable. While the six-month interval was selected to separate the measurement of predictor and outcome variables, we acknowledge the possibility of timing-related confounds due to organizational changes that may have occurred during this period. This highlights the need for future research to carefully consider the optimal time lag between data collection waves, balancing the benefits of temporal separation against the risk of introducing bias from unmeasured contextual events. Additionally, instead of relying solely on perceptual measures, future research could integrate objective performance indicators to enhance the validity of findings. Using a combination of subjective and objective data would help future studies reduce the influence of extraneous variables and more comprehensively assess task crafting’s impact on performance. Moreover, while this study treated affective commitment as a static predictor, future research should adopt a more dynamic perspective by capturing its temporal fluctuations. Longitudinal or experience-sampling designs could offer deeper insights into how affective commitment evolves over time and interacts with task crafting to influence employee performance.

Secondly, the current study included a relatively small number of groups (i.e., supervisors) and an uneven distribution of subordinates across them, with an average of 2.8 subordinates per supervisor. Although the sample size met the minimum requirements for multilevel modeling, future research should aim to secure a larger number of supervisor–subordinate dyads with more balanced groupings to enhance statistical power and the reliability of cross-level estimates. Additionally, collecting data from a wider range of industries would improve the generalizability and contextual robustness of the findings. A larger sample would also enable the inclusion of a broader set of control variables—such as hierarchical position, workload, and organizational culture—which were not incorporated in the present study due to concerns about model complexity and statistical power.

Thirdly, this study was conducted in South Korea. While our findings make both theoretical and practical contributions, cultural and institutional differences may influence how task crafting operates in different organizational contexts. Given that work behaviors and job redesign practices can vary across cultures, future studies should examine various countries and cultural environments to gauge the cross-cultural validity of our findings. A comparative approach could provide deeper insights into the generalizability of the findings and allow us to discover potential cross-cultural variations in the roles of affective commitment and strategic alignment. Additionally, our finding that female employees received higher task performance ratings than male employees may reflect sociocultural dynamics specific to the South Korean context. Factors such as increased performance pressure on women in male-dominated settings, survivor bias among high-performing female employees, or evolving gender norms in performance evaluations may have contributed to this outcome (Joshi et al., 2015; Roth et al., 2012). Future research should examine whether this gender effect is replicated in other cultural contexts, and how gender interacts with organizational norms and evaluator perceptions across countries.

Fourthly, this study focused on particularly on task crafting and investigated its relationship with task performance. However, relational crafting and cognitive crafting, which involve modifying social interactions at work and perceptions of work, respectively (Wrzesniewski & Dutton, 2001), may also play significant roles in shaping task performance. Accordingly, future research should explore how these dimensions of job crafting affect performance outcomes and identify the conditions under which they contribute to improving job effectiveness. During this it would be valuable to consider contextual variables such as job autonomy, organizational structure, and cultural norms, which may shape the extent to which employees can engage in different forms of crafting and how those efforts are evaluated within the organization.

Fifthly, while this study primarily considered affective commitment and strategic alignment as key contextual factors, other resource-based situational variables may influence task crafting effectiveness. Future research could explore how additional resources—such as job autonomy, social support, or career development opportunities—moderate the relationship between task crafting and task performance. Job autonomy, in particular, may serve as one of boundary condition as employees with greater discretion over their tasks are better positioned to craft their jobs in ways that align with organizational goals. Studies examining a broader range of situational variables could further refine the theoretical framework and provide a more comprehensive understanding of how proactive job modifications contribute to performance.

Finally, although this study explained how motivational and organizational resources interact to determine the effectiveness of task crafting by applying conservation of resources theory, incorporating additional theoretical perspectives could enhance our understanding of these dynamics. For example, in future studies it could be greatly helpful for self-determination theory or job demands-resources theory to play a role in accounting for the effectiveness of employees’ task crafting behavior, either individually or complementarily. Specifically, self-determination theory could provide insight into the motivational mechanisms that lead employees to participate in effective task crafting, and job demands-resources theory could highlight how different workplace conditions shape the availability of resources needed for effective task crafting. Future research incorporating several theoretical lenses would provide a richer and more holistic view of how employees actively modify their work to improve performance. In summary, these directions for future research could refine the theoretical foundations of task crafting and expand its practical applicability across different organizational and cultural contexts.

## 6. Conclusions

Beyond merely fulfilling assigned duties, members of organizations increasingly find themselves shaping the boundaries of their tasks. However, task modifications that are misaligned with organizational objectives or negatively impact task performance undermine the sustainability of such behavior. Focusing on the conditions that enable task crafters to become high performers, this study found that organizations need to foster environments where employees are both affectively committed to and strategically aligned with organizational goals. By ensuring that employees’ task modifications contribute meaningfully to organizational priorities, organizations can more effectively harness the benefits of task crafting. Ultimately, this study offers insights into how organizations can cultivate both proactive and effective behaviors among their members, leading to sustainable performance improvements.

## Figures and Tables

**Table 1:** Comparison of measurement models.

Model	Factor	*χ* ^2^	df	Δ*χ*^2^	CFI	IFI	RMSEA
Baseline model	4 factors: TC, AC, SA, TP	296.11	182		0.95	0.95	0.07
Model 1	3 factors: TC, (AC + SA), TP	545.63	185	249.52 ***	0.83	0.83	0.12
Model 2	3 factors: (TC + AC), SA, TP	625.55	185	329.14 ***	0.79	0.79	0.13
Model 3	3 factors: (TC + SA), AC, TP	624.25	185	328.44 ***	0.79	0.80	0.13
Model 4	2 factors: (TC + AC + SA), TP	904.34	187	608.23 ***	0.66	0.66	0.17
Model 5	1 factor: (TC + AC + SA + TP)	1336.64	188	1040.53 ***	0.46	0.46	0.21

Note: TC = Task crafting; AC = affective commitment; SA = strategic alignment; TP = task performance; RMSEA = root mean square error of approximation; CFI = comparative fit index; IFI = incremental fit index. *** *p* < 0.001.

**Table 2:** Means, standard deviations, and correlations.

Variables	M	SD	1	2	3	4	5	6	7	8
1. Age	39.83	10.15								
2. Gender	0.60	0.49	0.10							
3. Educational level	2.28	1.00	−0.10	−0.09						
4. Organizational tenure	5.95	5.96	0.33 ***	−0.02	−0.15					
5. Task crafting	3.29	0.64	0.10	0.07	0.24 **	0.22 **	(0.91)			
6. Affective commitment	3.56	0.73	0.15	0.06	−0.12	0.25 **	0.36 ***	(0.87)		
7. Strategic alignment	3.90	0.72	0.05	0.09	0.14	0.23 **	0.44 ***	0.51 ***	(0.91)	
8. Task performance	3.77	0.61	−0.03	−0.22 **	−0.03	−0.06	0.00	−0.11	−0.02	(0.92)

Note: *N* = 138. All tests are two-tailed. Reliabilities are on the diagonal in parentheses. ** *p* < 0.01; *** *p* < 0.001.

**Table 3:** Results of hierarchical linear modeling predicting task performance.

Variables	Model 1	Model 2	Model 3	Model 4	Model 5
Age	0.00	0.14	0.00	0.00	0.00
Gender	−0.30 **	−0.30 **	−0.30 **	−0.31 **	−0.33 **
Educational level	−0.04	−0.05	−0.05	−0.05	−0.05
Organizational tenure	−0.01	−0.01	−0.01	−0.01	−0.01
Task crafting	0.06	0.07	0.07	0.06	−0.02
Affective commitment		−0.12	−0.14	−0.08	−0.16
Strategic alignment		0.09	0.10	0.08	0.07
Task crafting × Affective commitment		0.17 *		0.23 *	0.22 *
Task crafting × Strategic alignment			0.15	0.10	0.15
Affective commitment × Strategic alignment				−0.18 *	−0.06
Task crafting × Affective commitment × Strategic alignment					0.19 *
Pseudo *R*^2^	0.02	0.05	0.05	0.05	0.07

Note: *N* = 138. All tests are two-tailed. Values are standardized regression coefficients. * *p* < 0.05; ** *p* < 0.01.

## Data Availability

The data supporting the findings of this study are available from the corresponding author upon reasonable request.
